# Single-nephron assessment of urate excretion in patients with IgA nephropathy

**DOI:** 10.1093/ckj/sfae036

**Published:** 2024-02-12

**Authors:** Hirokazu Marumoto, Nobuo Tsuboi, Takaya Sasaki, Yusuke Okabayashi, Kotaro Haruhara, Go Kanzaki, Kimiyoshi Ichida, Takashi Yokoo

**Affiliations:** Division of Nephrology and Hypertension, Department of Internal Medicine, The Jikei University School of Medicine, Tokyo, Japan; Division of Nephrology and Hypertension, Department of Internal Medicine, The Jikei University School of Medicine, Tokyo, Japan; Division of Nephrology and Hypertension, Department of Internal Medicine, The Jikei University School of Medicine, Tokyo, Japan; Division of Nephrology and Hypertension, Department of Internal Medicine, The Jikei University School of Medicine, Tokyo, Japan; Division of Nephrology and Hypertension, Department of Internal Medicine, The Jikei University School of Medicine, Tokyo, Japan; Division of Nephrology and Hypertension, Department of Internal Medicine, The Jikei University School of Medicine, Tokyo, Japan; Department of Pathophysiology, Tokyo University of Pharmacy and Life Sciences, Tokyo, Japan; Chiba Health Promotion Center, East Japan Railway Company, Chiba, Japan; Division of Nephrology and Hypertension, Department of Internal Medicine, The Jikei University School of Medicine, Tokyo, Japan

To the Editor,

Hyperuricemia and abnormal urate excretion are thought to be involved in the progression of chronic kidney disease (CKD). Among the causative diseases of CKD, immunoglobulin A nephropathy (IgAN) has been the most extensively studied in relation to blood urate levels [1, 2]. Although hyperuricemia was initially considered a poor prognostic factor for IgAN, recent large-scale studies have reported its limited sensitivity in certain populations, including men, the elderly and patients with an impaired kidney function or advanced tubulointerstitial injury (S1, S2).

We recently established a method to estimate the total nephron number in clinical practice and reported nephron number estimates in patients with IgAN [3]. As a subanalysis of our data, we estimated the urinary urate excretion per nephron in patients with IgAN. Single-nephron urate excretion was calculated by dividing the total urinary urate excretion by the number of non-globally sclerotic glomeruli in both kidneys and was compared among CKD stage groups. The detailed methods for patient selection and nephron number measurements are described in the Supplementary Methods.

This retrospective study included 158 adult Japanese patients who underwent native kidney biopsy and who were diagnosed with primary IgAN at Jikei Hospital, Tokyo, between 2007 and 2017. The clinicopathological and morphometric findings of the patients are summarized in Supplementary data, Table S1. The mean age was 39.0 years, and 95 (60.5%) patients were male. As CKD stages advance, patients tend to be older, and to have more systemic hypertension, heavier proteinuria and more severe chronic histopathological lesions, including interstitial fibrosis/tubular atrophy. In the CKD stages G1, G2, G3a, G3b and G4–5 groups, the average single-nephron urate excretion was 0.25, 0.28, 0.38, 0.79 and 0.74 ng/mg creatinine, respectively. Figure 1 shows the fold-differences in uric acid–related parameters among the CKD stage groups relative to CKD stage G1 as the reference. In this study population, conventionally used uric acid–related parameters, such as serum urate, total urinary urate excretion and fractional excretion of urate, showed only modest changes with advancing CKD stages. In contrast, single-nephron urate excretion was markedly increased in patients with stage G3b or more advanced CKD stages, even though compensatory excretion from the intestinal tract is expected to increase in these patients (S3, S4).

**Figure 1:**
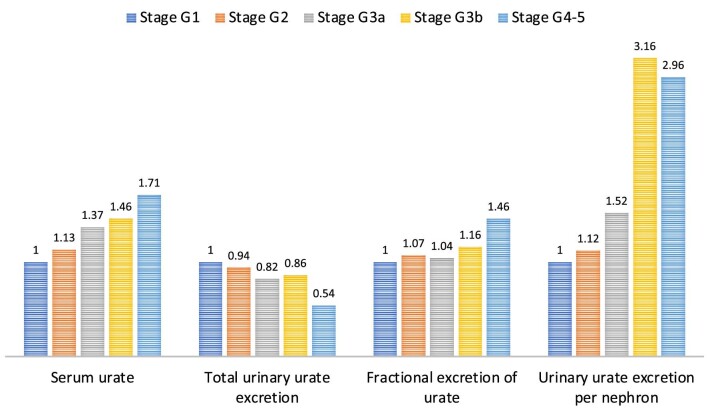
Fold differences in uric acid–related parameters among CKD stage groups. Differences between CKD stage groups are shown for uric acid–related parameters, including the serum urate level, total urinary urate excretion, fractional excretion of urate and urinary urate excretion per nephron. For each parameter, the values relative to CKD stage G1 are shown.

A biopsy study showed a close relationship between serum urate levels in patients with IgAN and periglomerular arteriolar hyalinosis, suggesting that serum urate disrupts the arteriolar autoregulatory function and induces glomerular hyperperfusion injury (S5). Another clinical study demonstrated that hyperuricemia was independently associated with the progression of tubulointerstitial lesions in patients with IgAN [4]. However, the mechanisms by which urate exposure to tubules promotes kidney disease progression remain to be fully elucidated. A previous study showed that urate induces epithelial–mesenchymal transition of kidney tubular cells *in vitro* in a dose-dependent manner [5]. This suggests that urate exposure to tubules may induce direct cell injury under certain circumstances in which urate is abnormally and constitutively concentrated in the tubulointerstitium. The elevated single-nephron urate levels in IgAN patients with advanced CKD may represent part of the pathophysiological abnormalities of urate dynamics in the tubulointerstitium. A major limitation of this cross-sectional study was the difficulty in referring to causal relationships. Further studies, including validation of progressive kidney diseases other than IgAN, are warranted.

In conclusion, we evaluated urinary urate excretion per nephron in IgAN patients and showed markedly concentrated levels of single-nephron urate in patients with advanced CKD. Clinical assessment of single-nephron excretion of a certain molecule, as demonstrated for urate in this study, may be a promising tool for elucidating the pathophysiology of human kidney diseases, such as IgAN, in which nephron mass reduction is a common feature of disease progression.

## Supplementary Material

sfae036_Supplemental_File
