# Supplementary material for: Single-nephron assessment of urate excretion in patients with IgA nephropathy
Source: Clin Kidney J. 2024 Feb 12;17(3):sfae036. doi: 10.1093/ckj/sfae036 (PMC10913940; doi:10.1093/ckj/sfae036)
Supplement: sfae036_Supplemental_File [file sfae036_supplemental_file.pdf]

Supplemental Materials

Supplemental Methods

Patient selection

Diagnosis of IgA nephropathy was based on typical kidney biopsy features of mesangial proliferative glomerulonephritis, the presence of dominant or co-dominant glomerular IgA deposition by immunohistochemistry or immunofluorescence, and the presence of electron dense mesangial deposits by electron microscopy. Patients with other systemic diseases associated with glomerular IgA deposition, including IgA vasculitis, liver cirrhosis, and systemic lupus erythematosus were excluded. For this study, exclusion criteria were applied as follows: (i) patients with under 18 years old, (ii) patients treated with urate-lowering drugs, (iii) patients whose computed tomography (CT) images were not available within 1 year before kidney biopsy, (iv) patients whose kidney biopsy specimens contained < 5 non-globally sclerotic glomeruli on light microscopy or a cortical area < 2 mm^2 and (v) patients with no critical data for analysis available. Consent was obtained by opting out for individual participants. All participants were provided the opportunity to ask questions and discuss the study. This study was approved by the ethics review board of the Jikei University School of Medicine [30-385 (9406)]

Definition

Hypertension was defined as a systolic blood pressure of ≥140 mmHg, a diastolic blood pressure of ≥90 mmHg, or the use of antihypertensive medications. The estimated glomerular filtration rate (eGFR) was calculated from serum creatinine using a modified equation for GFR based on Japanese individuals: $eGFR = 194 \times age^{-0.287} \times (serum\ creatinine)^{-1.094}$ (×0.739 if female) (S6). Chronic kidney disease (CKD) stages were defined based on eGFR for Japanese individuals and were classified into 5 categories as follows: CKD stage G1: ≥90, stage G2: 60 to 89, stage G3a: 45 to 59, stage G3b: 30 to 44, and stage G4–5: <30 mL/min/1.73m^2, respectively. Urinary protein excretion was measured using spot urine and presented as urinary protein to creatinine ratio. Parameters related to urate handling of the kidney, including serum urate level, total urinary urate excretion, fractional excretion of urate and single-nephron urate excretion per nephron, were examined. Urate excretion was measured using spot urine and presented as urinary urate to creatinine ratio.

Pathological analysis

All kidney tissue specimens were obtained by percutaneous needle biopsy. The tissues were embedded in paraffin, cut into 3 μm sections, and stained with hematoxylin-eosin, periodic acid-Schiff, Masson's trichrome, and periodic acid silver-methenamine. All biopsy samples were stained by immunohistochemistry or immunofluorescence for IgG, IgA, IgM, C3 and C1q. Globally sclerosed glomeruli was defined as the entire glomerulus involved by sclerosis. Non-globally sclerotic glomerulus

was used when there was no sclerosis or sclerosis only involved part of the glomerulus. Glomeruli containing segmental scars or crescents were included among non-globally sclerotic glomerulus. The cortical area with interstitial fibrosis/tubular atrophy was semi-quantitatively scored to the nearest 10% and average values were estimated across the entirety of each biopsy specimen. Arteriosclerotic lesions and arteriolar hyalinosis were graded as previously described (S7). The Oxford scores for mesangial hypercellularity (M), endocapillary hypercellularity (E), segmental sclerosis or adhesion (S), interstitial fibrosis and tubular atrophy (T) and crescents (C) were determined as described previously (S8).

Morphological measurements

The thickness of all the obtained CT images was 5.0 mm. Kidney parenchymal volumes were measured as previously described using software (ITK-SNAP version 3.6, University of Pennsylvania, Philadelphia, PA, www.itksnap.org) to semi-automatically segment the parenchymal images obtained from unenhanced CT images of both kidneys (S9). Estimated kidney cortical volumes were calculated using an equation as follows: estimated cortical volume (cm^3) = -1.3 (intercept) + 0.71 × parenchymal volume (cm^3) (S9). Kidney biopsies were semi-automatically analyzed to measure the individual areas of all glomerular capillary tufts and the total area of the obtained kidney cortex using image analysis software (Win roof 2017, Mitani Corporation, Tokyo, Japan). Glomerular area was defined as an averaged area described by outer capillary loops of the tuft. Mean glomerular volume was calculated from the measured glomerular area as follows: Mean glomerular volume = $\frac{\beta}{d} \times (\text{mean glomerular area})^{\frac{3}{2}}$, where β is a dimensionless shape coefficient (β = 1.382), and d is a size distribution coefficient (d = 1.01) (S10). The volumetric density of non-globally sclerotic glomeruli was determined using the Weibel-Gomez stereological method as follows: NSG density = $\frac{1}{\beta} \times \sqrt[2]{\frac{\left(\frac{\text{Total number of glomerulus}}{\text{Area of cortex}}\right)^3}{\frac{\text{Total area of glomerulus}}{\text{Area of cortex}}}}$, where β is a dimensionless shape coefficient (β = 1.38) (S11). The total number of non-globally sclerotic glomeruli per kidney was calculated by multiplying the estimated cortical volume and the volumetric non-sclerotic glomerular density (S12). The calculated value was divided by 2 for per kidney, by 1.43 for correcting tissue volume shrinkage due to paraffin embedding, and by 1.268 for correcting volume shrinkage due to loss of tissue perfusion pressure (S12). Urinary uric acid excretion per nephron was defined by dividing total uric acid excretion by the estimated non-globally sclerotic glomerular number of both kidneys.

Statistical analyses

Patients' characteristics at baseline are presented as mean (standard deviation [SD]) or median [25th–75th percentile] for continuous variables, and frequencies and proportions for categorical variables. The Mann–Whitney U test was used to compare continuous variables between two groups. For three or more groups, trends were tested using linear regression or Jonckheere–Terpstra test. All reported p values were

two-sided. P values of <0.05 were considered to be statistically significant. All statistical analyses were performed using EZR (Saitama Medical Center, Jichi Medical University), a graphical user interface for R (R Foundation for Statistical Computing, version 3.5.2) (S13).

Supplemental References

S1 Nagasawa Y, Yamamoto R, Shoji T, Shinzawa M, Hasuike Y, Nagatoya K, Yamauchi A, Hayashi T, Kuragano T, Moriyama T, Isaka Y, Nakanishi T. Serum Uric Acid Level Predicts Progression of IgA Nephropathy in Females but Not in Males. PLoS One. 2016 Aug 25;11(8):e0160828.

S2 Zhu B, Yu DR, Lv JC, Lin Y, Li Q, Yin JZ, Du YY, Tang XL, Mao LC, Li QF, Sun Y, Liu L, Li XF, Fei D, Wei XY, Zhu CF, Cheng XX, Chen HY, Wang YJ. Uric Acid as a Predictor of Immunoglobulin A Nephropathy Progression: A Cohort Study of 1965 Cases. Am J Nephrol. 2018;48(2):127-136.

S3 Ohashi Y, Toyoda M, Saito N, Koizumi M, Kanai G, Komaba H, Kimura M, Wada T, Takahashi H, Takahashi Y, Ishida N, Kakuta T, Fukagawa M, Ichida K. Evaluation of ABCG2-mediated extra-renal urate excretion in hemodialysis patients. Sci Rep. 2023 Jan 13;13(1):93.

S4 Yano H, Tamura Y, Kobayashi K, Tanemoto M, Uchida S. Uric acid transporter ABCG2 is increased in the intestine of the 5/6 nephrectomy rat model of chronic kidney disease. Clin Exp Nephrol. 2014 Feb;18(1):50-5.

S5 Kohagura K, Kochi M, Miyagi T, Zamami R, Nagahama K, Yonemoto K, Ohya Y. Augmented Association Between Blood Pressure and Proteinuria in Hyperuricemic Patients With Nonnephrotic Chronic Kidney Disease. Am J Hypertens. 2018 Mar 10;31(4):480-485.

S6 Matsuo S, Imai E, Horio M, et al. Revised Equations for Estimated GFR From Serum Creatinine in Japan. *Am J Kidney Dis* 53(6): 982–992, 2009

S7 Roberts ISD, Cook HT, Troyanov S, et al. The Oxford classification of IgA nephropathy: Pathology definitions, correlations, and reproducibility. *Kidney Int* 76(5):546–556, 2009

S8 Coppo R, Troyanov S, Bellur S, et al. Validation of the Oxford classification of IgA nephropathy in cohorts with different presentations and treatments. *Kidney Int* 86(4): 828–836, 2014

S9 Sasaki T, Tsuboi N, Kanzaki G, et al. Biopsy-based estimation of total nephron number in Japanese living kidney donors. *Clin Exp Nephrol* 23(5): 629–637, 2019

S10 Weibel E, Gomez D. A principle for counting tissue structures on random sections. *J Appl Physiol* 17(2): 343–348, 1962

S11 Fulladosa X, Moreso F, Narváez JA, Grinyó JM, Serón D. Estimation of total glomerular number in stable renal transplants. *J Am Soc Nephrol* 14(10): 2662–2668, 2013

S12 Denic A, Lieske JC, Chakkera HA, et al. The Substantial Loss of Nephrons in Healthy Human Kidneys with Aging. *J Am Soc Nephrol* 28(1): 313–320, 2016

S13 Kanda Y. Investigation of the freely available easy-to-use software “EZR” for medical statistics. *Bone Marrow Transplant* 48(3): 452–458, 2013

Supplemental Table S1. Comparison of clinical, histopathological and morphometrical findings between CKD stage groups

	CKD G1 (n=20)	CKD G2 (n=74)	CKD G3a (n=32)	CKD G3b (n=14)	CKD G4, 5 (n=18)	P for trend
**Clinical findings**						
Age (years)	28.0 ± 4.9	38.5 ± 11.8	45.5 ± 10.8	46.1 ± 12.2	50.8 ± 15.6	< 0.001
Male, n (%)	12 (60.0)	40 (54.1)	23 (71.9)	7 (50.0)	14 (77.8)	0.21
Body mass index (kg/m^2)	21.5 ± 4.5	22.7 ± 3.8	23.5 ± 3.4	24.3 ± 4.6	23.2 ± 2.9	0.06
Hypertension, n (%)	2 (10.0)	14 (18.9)	3 (9.4)	3 (21.4)	9 (50.0)	0.008
RAAS inhibiter use, n (%)	10 (50.0)	49 (66.2)	29 (90.6)	13 (92.9)	18 (100.0)	< 0.001
Diuretic use, n (%)	0 (0.0)	1 (1.4)	1 (3.1)	2 (14.3)	1 (5.6)	0.12
HbA1c (%)	5.4 ± 0.7	5.4 ± 0.3	5.5 ± 0.3	5.5 ± 0.4	5.5 ± 0.4	0.10
Serum albumin (mg/dL)	4.2 ± 0.3	3.9 ± 0.5	3.8 ± 0.4	3.7 ± 0.7	3.3 ± 0.5	< 0.001
Serum urate (mg/dL)	5.2 ± 1.3	5.9 ± 1.3	7.1 ± 1.0	7.6 ± 1.1	8.9 ± 1.5	< 0.001
Serum creatinine (mg/dL)	0.68 ± 0.12	0.83 ± 0.14	1.12 ± 0.13	1.50 ± 0.26	2.38 ± 0.55	< 0.001
eGFR (mL/min/1.73m^2)	104.3 ± 16.1	75.2 ± 8.5	53.5 ± 4.6	36.8 ± 3.3	24.0 ± 4.1	< 0.001
Urinary creatinine (mg/dL)	37.4 ± 18.4	35.0 ± 19.0	30.7 ± 11.6	32.0 ± 20.9	20.3 ± 10.4	0.01
Urinary protein (g/g creatinine)	0.51 [0.33−0.64]	0.58 [0.40−0.97]	1.14 [0.42−1.56]	0.88 [0.67−2.02]	2.15 [1.66−3.28]	< 0.001
Urinary RBC count, grade 1-5*, n (%)	13 (65.0)	62 (83.6)	30 (93.8)	10 (71.4)	12 (66.7)	0.53
Urinary urate (mg/g creatinine)	0.41 ± 0.12	0.41 ± 0.11	0.36 ± 0.08	0.33 ± 0.12	0.31 ± 0.11	0.001
Fractional excretion of urate (%)	5.6 ± 2.2	6.0 ± 2.1	5.8 ± 1.3	6.5 ± 2.1	8.2 ± 3.2	0.001
Single-nephron urate excretion (ng/g creatinine)	0.25 ± 0.17	0.28 ± 0.16	0.38 ± 0.25	0.79 ± 0.54	0.74 ± 0.57	< 0.001
**Histopathological findings**						
Total glomeruli identified in biopsy (%)	26.4 ± 13.0	25.4 ± 11.8	22.6 ± 10.9	16.5 ± 6.9	18.3 ± 8.0	0.01
Glomeruli with global glomerulosclerosis (%)	6.5 ± 7.6	8.9 ± 8.4	19.6 ± 15.0	23.7 ± 15.4	35.10 ± 18.2	< 0.001
Atherosclerotic lesion, grade 1-2 †, n (%)	3 (15.0)	39 (52.7)	21 (65.6)	12 (85.7)	15 (83.3)	< 0.001
Arterial hyaline, grade 1-3 † †, n (%)	4 (20.0)	23 (31.1)	12 (37.5)	9 (64.3)	9 (50.0)	0.052
Interstitial fibrosis/tubular atrophy (%)	8.8 ±7.4	9.7 ±7.1	17.7 ±14.1	31.1 ±19.6	42.8 ±19.9	< 0.001
Oxford score						
Patients with M1, n (%)	8 (40.0)	33 (44.6)	15 (46.9)	5 (35.7)	11 (61.1)	0.63
Patients with E1, n (%)	1 (5.0)	13 (17.6)	4 (12.5)	1 (7.1)	6 (33.3)	0.13
Patients with S1, n (%)	17 (85.0)	62 (83.8)	31 (96.9)	13 (92.9)	17 (94.4)	0.29
Patients with T1+2, n (%)	1 (5.0)	3 (4.1)	7 (21.9)	7 (50.0)	14 (77.8)	< 0.001
Patients with C1+2, n (%)	6 (30.0)	27 (36.5)	15 (46.9)	6 (42.9)	9 (50.0)	0.62
**Morphometric findings**						
Cortical volume (cm^3/kidney)	107.5 ± 21.4	97.8 ± 17.6	92.8 ± 16.9	74.5 ± 17.1	75.6 ± 23.2	< 0.001
Non-globally sclerotic glomerular density (/mm^3)	17.8 ± 6.8	16.4 ± 6.5	11.9 ± 4.8	7.6 ± 4.5	6.9 ± 2.9	< 0.001
Non-globally sclerotic glomeruli (x10^4/kidney)	105.9 ± 47.9	87.3 ± 35.1	61.0 ± 25.7	30.8 ± 20.1	28.8 ± 17.6	< 0.001

Abbreviations: CKD, chronic kidney disease; RAAS, renin-angiotensin aldosterone system; GFR, glomerular filtration rate; RBC, red blood cell.

Footnote: Values are presented as the means ± standard deviations or median [25th–75th percentile].

* The urinary RBC count was graded as follows: grade 0, <5/high power field (HPF); grade 1, 5–9/HPF; grade 2, 10–19/HPF; grade 3, 20–49/HPF; grade 4, 50–99/HPF; and grade 5, >99/HPF.

†Arteriosclerotic lesions were defined as normal (grade 0), and less than 50% (grade 1) or more than 50% of the thickness of media (grade 2).

††Arteriolar hyaline was graded as the proportion of arterioles affected (grade 0, <1%; grade 1, 1–25%; grade 2, 26–50%; grade 3, >50%).
